# Oviposition responses of *Aedes* mosquitoes to bacterial isolates from attractive bamboo infusions

**DOI:** 10.1186/s13071-015-1068-y

**Published:** 2015-09-23

**Authors:** Loganathan Ponnusamy, Coby Schal, Dawn M. Wesson, Consuelo Arellano, Charles S. Apperson

**Affiliations:** Department of Entomology and W. M. Keck Center for Behavioral Biology, North Carolina State University, Campus Box 7613, Raleigh, NC 27695-7613 USA; Department of Tropical Medicine, Tulane Health Sciences Center, Tulane University, New Orleans, LA 70112 USA; Department of Statistics, North Carolina State University, Raleigh, NC 27695 USA

**Keywords:** *Aedes aegypti*, *Aedes albopictus*, Bacterial isolates, Behavioral assays, Attractants

## Abstract

**Background:**

The mosquitoes *Aedes aegypti* and *Aedes albopictus* are vectors of pathogenic viruses that cause major human illnesses including dengue, yellow fever and chikungunya. Both mosquito species are expanding their geographic distributions and now occur worldwide in temperate and tropical climates. Collection of eggs in oviposition traps (ovitraps) is commonly used for monitoring and surveillance of container-inhabiting *Aedes* populations by public health agencies charged with managing mosquito-transmitted illness. Addition of an organic infusion in these traps increases the number of eggs deposited. Gravid females are guided to ovitraps by volatile chemicals produced from the breakdown of organic matter by microbes.

**Methods:**

We previously isolated and cultured 14 species of bacteria from attractive experimental infusions, made from the senescent leaves of canebrake bamboo (*Arundinaria gigantea*). Cultures were grown for 24 h at 28 °C with constant shaking (120 rpm) and cell densities were determined with a hemocytometer. Behavioral responses to single bacterial isolates and to a mix of isolates at different cell densities were evaluated using two-choice sticky-screen bioassay methods with gravid *Ae. aegypti* and *Ae. albopictus.*

**Results:**

In behavioral assays of a mix of 14 bacterial isolates, significantly greater attraction responses were exhibited by *Ae. aegypti* and *Ae. albopictus* to bacterial densities of 10^7^ and 10^8^ cells/mL than to the control medium. When we tested single bacterial isolates, seven isolates (B1, B2, B3, B5, B12, B13 and B14) were significantly attractive to *Ae. aegypti*, and six isolates (B1, B5, B7, B10, B13 and B14) significantly attracted *Ae. albopictus.* Among all the isolates tested at three different cell densities, bacterial isolates B1, B5, B13 and B14 were highly attractive to both *Aedes* species.

**Conclusions:**

Our results show that at specific cell densities, some bacteria significantly influence the attraction of gravid *Ae. aegypti* and *Ae. albopictus* females to potential oviposition sites. Attractive bacterial isolates, when formulated for sustained release of attractants, could be coupled with an ovitrap containing a toxicant to achieve area-wide management of *Aedes* mosquitoes.

## Background

*Aedes* (*Stegomyia*) *aegypti* L. and *Ae.* (*Stegomyia*)* albopictus* (Skuse) are the principal mosquito vectors of dengue fever, yellow fever and chikungunya viruses on a global basis [1]. These *Stegomyia* mosquitoes are day-active and lay eggs in domestic water-filled containers [2]. Previous studies have shown that the oviposition behavior of mosquitoes is mediated by a combination of visual, olfactory, tactile, and chemo-tactile cues associated with their container habitats [3–5]. Many of the chemical cues mediating oviposition behavior originate from microbial fermentation in water containers [6–11]. Specifically, microbial metabolites act as oviposition attractants and/or stimulants for various species of mosquitoes [9, 12, 13]. Infusions produced from sterilized plants and water elicited significantly diminished oviposition responses [14], evidencing the essential role of microbes in the production of oviposition attractants.

Only a few studies have evaluated the response of mosquitoes to pure cultures of bacteria. Positive oviposition responses were exhibited by gravid *Aedes aegypti* and *Culex quinquefasciatus* to bacteria isolated from hay infusion [15]. In a bioassay of bacterial species, gravid *Cx. quinquefasciatus* oviposited significantly more egg rafts in cups that contained agar washes of *Enterobacter agglomerans *(Beijerinck), *Pseudomonas maltophilia* (Hugh), and *Bacillus cereus* (Franklin) than in control cups containing water only [16]. Likewise, *Acinitobacter calcoaceticus* (Beijerinck) and *Enterobacter cloacae* (Jordan) isolated from larval-rearing water attracted gravid *Ae. aegypti* [6], and gravid *Ae. aegypti* exhibited positive oviposition responses to several species of bacteria isolated from the larval habitat of *Culex* mosquitoes [17]. In the laboratory, *Ae. aegypti* and *Ae. albopictus* exhibited positive oviposition responses to *Bacillus cereus* isolated from an unknown source [18]*.* Recent reviews also describe the use of microbial volatiles by mosquitoes as chemical cues to locate oviposition sites containing nutrient resources [19, 20].

In a recent study*,* we reported that a mixture of 14 bacterial species isolated from an experimental infusion of senescent leaves of canebrake bamboo (*Arundinaria gigantea*) was highly attractive to gravid *Ae. aegypti* and *Ae. albopictus* [21]. Here, we describe the behavioral responses of gravid females of both mosquito species to different cell densities of a mixture of the 14 bacterial species and to single-isolate cultures*.* In this paper, our objective was to evaluate individual bacteria isolates from canebrake bamboo leaf infusion as oviposition attractants for gravid mosquitoes and to determine the optimal cell concentration of the isolates eliciting maximum attraction. This approach has allowed us to select the four most attractive bacteria isolates and we have formulated the bacteria using an alginate encapsulation method. We have completed field-testing of a lethal ovitrap combined with the bacterial beads in Iquitos, Peru. An analysis of the data derived from this field trial and manuscript describing our research findings on formulation of attractive bacteria and the field trial of the lethal ovitrap in controlling *Ae. aegypti* is in preparation.

## Methods

### Origin and maintenance of mosquitoes

*Aedes aegypti* and *Ae. albopictus* colonies were established from field-collected eggs from New Orleans, LA in 2003*.* At 6–8 month intervals, adults reared from freshly field-collected eggs were added to each mosquito colony to maintain genetic diversity. Larvae were reared as described by Trexler *et al.* [13]. Briefly, mosquito colonies were maintained in an insectary at ~28 °C, ~75 % RH, and a photoperiod of 14 h light:10 h dark, including two twilight periods (60 min each). Eggs for maintenance of mosquito colonies were obtained from females that were blood fed on a human forearm.

### Bioassay methods

To assess olfactory attraction, we used a sticky screen bioassay to differentiate responses to volatile chemicals that guide mosquitoes to an oviposition site from egg-laying due to chemical cues that arrest and stimulate mosquitoes to oviposit, as described by Ponnusamy *et al.* [9, 14]. Briefly, bioassays were conducted in Plexiglas® cages (30 x 30 x 30 cm) fitted with stockinet cloth sleeves. In each cage, two 125-mL polypropylene cups, filled with 30 mL of either a test bacterial isolate or control medium, were placed randomly in diagonal corners of the bioassay cage. A disc of glue-coated galvanized screen was suspended below the lip of each cup. Following placement of the cups, 10 gravid females were transferred into the cage. After a 24-h test period, the numbers of females stuck on each screen were counted and a percentage (based on the total number responding) was calculated for the test and control cups.

### Source of bacterial isolates and growth conditions

The bacterial isolates used in this study had been previously cultured from bioactive canebrake bamboo (*Arundinarea gigantea*) leaf infusion using the R2A medium of Reasoner and Geldreich [22]. The 14 isolates listed in Table 1 were purified and identified to species as described by Ponnusamy *et al.* [9]. Recently, we showed that odorants produced by the mixture of these 14 species attracted gravid *Ae. aegypti* [21]. Subsequently, we developed a modified R2A medium (MR2A) to obtain optimal growth of bacterial isolates. The new MR2A liquid medium contained 1000 mg/L of skim milk (Difco), 500 mg/L dextrose (Sigma), 50 mg/L yeast extract (Fisher), 50 mg/L peptone (Fisher), 500 mg/L soluble starch (Fisher), 100 mg/L sodium pyruvate (Fisher), 50 mg/L casamino acids (Difco), 50 mg/L sodium chloride (Sigma), 100 mg/L magnesium sulfate (Fisher), and 300 mg/L dipotassium phosphate (Sigma) at pH 7.2. The modified MR2A medium was used in bioassays of bacterial isolates.

**Table 1:** Identification of bacterial species isolated from canebrake bamboo (*Arundinaria gigantea*) leaf infusions

Isolate^a^	Number of bases used to establish identity	Accession number in GenBank	Species corresponding to closest relative	Phylogenetic affiliation
			(% sequence identity)	
B1	714	EU341308	*Bacillus thuringiensis* (99)	*Firmicutes*
B2	617	EU341309	*Enterobacter asburiae* (98)	*Gammaproteobacteria*
B3	760	EU341310	*Enterobacter cancerogenus* (98)	*Gammaproteobacteria*
B4	758	EU341311	*Pseudomonas fulva* (99)	*Gammaproteobacteria*
B5	763	EU341312	*Lactococcus lactis* (99)	*Firmicutes*
B6	743	EU341313	*Enterobacter gergoviae* (97)	*Gammaproteobacteria*
B7	770	EU341314	*Enterobacter ludwigii* (97)	*Gammaproteobacteria*
B8	783	EU341315	*Klebsiella oxytoca* (98)	*Gammaproteobacteria*
B9	770	EU341316	*Klebsiella granulomatis* (98)	*Gammaproteobacteria*
B10	716	EU341319	*Pseudomonas plecoglossicida* (99)	*Gammaproteobacteria*
B11	770	EU341318	*Rhizobium huautlense* (97)	*Alphaproteo bacteria*
B12	604	EU341319	*Shigella dysenteriae* (76)	*Gammaproteobacteria*
B13	764	EU341320	*Citrobacter freundii* (97)	*Gammaproteobacteria*
B14	511	EU341321	*Brevundimonas vesicularis* (98)	*Alphaproteobacteria*

^a^Bacterial species were isolated in a previous investigation (Ponnusamy *et al.* 2008)

### Bioassay of the mixture of cultured bacteria

Bacterial cells (10^4^ cells per mL) of each of the 14 isolates were mixed, then 100 μL of this suspension was inoculated into 100 mL of MR2A medium and grown for 24 h. Bacterial cells from these cultures were used in density-response sticky screen attraction bioassays [9, 14]. A hemocytometer was used to estimate bacterial cell densities in MR2A cultures, which were serially diluted (10-fold) with sterile water to achieve final cell densities of 10^6^ to 10^9^ cells/mL in the 30 mL volumes contained in test bioassay cups. MR2A medium (without bacteria) was added to control cups after dilution with sterile water.

### Mosquito response to single bacteria isolates

Bacterial isolates were grown separately in MR2A medium at 28 °C on an orbital shaker (120 rpm) for 24 h. Bacterial cells were diluted to final concentrations of 10^8^, 10^7^, and 10^6^ cells/mL, determined with a hemocytometer. After dilution with sterile water, 30 mL of a given cell density of an isolate was added to the test cup. Similarly MR2A medium was diluted and added to the control cup. After a 24 h test period each bioassay was terminated and the number of females responding was recorded as described above.

Each bioassay trial included six cages per bacterial cell density and all three cell densities for a bacterial species were tested on the same day and at the same time. Three trials were completed for each bacterial species giving a total of 18 bioassays completed per cell density of each bacterial species.

### Data analyses

In each experiment, our null hypothesis assumed that each mosquito would select randomly between the ‘test-cup’ and ‘control-cup’ independently of other mosquitoes’ selection. The null hypothesis of no treatment effect was tested at a significance level of α = 0.05. Non-responders (free mosquitoes) were excluded from the test of the hypothesis. To determine if the responses of gravid mosquitoes to a treatment differed from their responses to a control, the total number of mosquitoes that were trapped on the test and control screens in each cage were analyzed using multinomial regression (PROC *GLIMMIX* [23]). We used a cut-off value of *P* = 0.05 for the False Discovery Rate procedure (PROC MULTTEST) to protect against Type I Error when testing multiple null hypotheses [24]. In other words, the number of tests that we employed did not significantly increase chances of making a type I error. Statistical analyses of response data were performed using SAS® software (version 9.4, SAS Institute; Cary, NC). After statistical analyses were completed, the response data were converted to percentages for graphical presentation of results.

### Ethical approval

The protocol for blood feeding was approved by the Biosafety Committee of North Carolina State University (Registration #2010-040421).

## Results

### Response of gravid mosquitoes to a mix of bacterial isolates

We evaluated the responses of gravid females of two mosquito species to four different cell densities of a mixture of 14 bacterial species that we had isolated previously from water infusions of canebrake bamboo leaves (Table 1). *Ae. aegypti* females exhibited significant attraction to 10^7^ and 10^8^ cells/mL (*P* < 0.01), but were significantly repelled by 10^9^ cells/mL (*P* = 0.0015). Similarly, significantly more gravid *Ae. albopictus* were attracted to bioassay cups containing 10^7^ and 10^8 ^cells/mL (*P* < 0.01) than to cups containing control medium, but *Ae. albopictus* females were neither attracted nor repelled by 10^9^ cells/mL (*P* = 0.2664) (Table 2).

**Table 2:** Results of sticky-screen bioassays of *Ae. aegypti* and *Ae. albopictus*, showing mean attraction and repellent responses to various cell densities of a mix of 14 bacterial species

Density (cells/mL)	No. of assays^a^	% of responders		SE^b^(%)	*t-*value	*P*-value^c^	Not responding % (±SE^b^)
		Treatment	Control				
*Ae. aegypti*							
10^9^	18	32	68	8.4	−3.78	0.0015	35 (9.6)
10^8^	18	74	26	7.3	5.31	0.0001	22 (7.3)
10^7^	18	72	28	7.3	4.99	0.0001	20 (6.1)
10^6^	18	55	45	8.5	1.05	0.3080	28 (7.1)
*Ae. albopictus*							
10^9^	18	55	45	7.95	1.15	0.2664	18 (6.6)
10^8^	18	72	28	6.99	5.39	0.0001	11 (3.7)
10^7^	18	68	32	7.31	4.29	0.0005	11 (4.7)
10^6^	18	54	46	8.31	0.85	0.4069	22 (5.8)

^a^Each assay consisted of 10 gravid females

^b^SE = Error represents half-width of a 95 % confidence intervals of the mean (SEM X 1.96)

^c^Significant *P*-value (*P* < 0.05) with positive *t*-value indicates attraction, whereas a negative *t*-value indicates repellence

Among the 4 different cell densities tested, non-responding *Ae. aegypti* females ranged between 20 and 35 %, and 11 to 22 % of *Ae. albopictus* females were not trapped and remained free in the bioassay arena (Table 2).

### Responses of *Aedes aegypti* to individual bacterial isolates

**Fig. 1:**
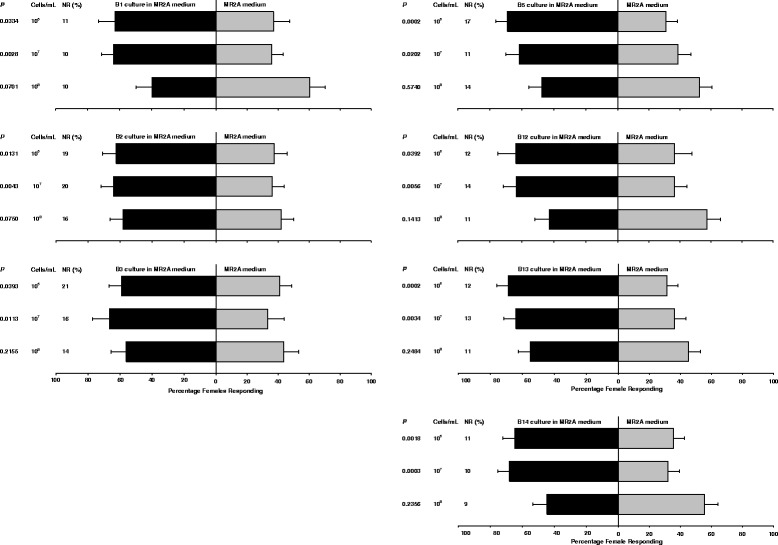
Results of 2-choice sticky screen attraction bioassays in which different bacterial species in MR2A liquid medium were tested for their attractiveness to *Ae. aegypti* against plain MR2A medium. Bars show the mean percentage of gravid females trapped on the sticky screens. Error bars represent half-width of a 95 % confidence intervals of the mean (SEM X 1.96). Each test consisted of 18 assays with 10 gravid females per assay. Bacterial isolates that did not elicit significant responses to any of the three cell densities and those that elicited significant responses to only one cell density are not shown in this figure. NR = not responding

Behavioral assays of each bacterial isolate indicated that seven isolates (B1, B2, B3, B5, B12, B13 and B14; see Table 1) elicited statistically significant attraction at two cell densities (Fig. 1, Table 3) . With these isolates, gravid mosquitoes were attracted to bacteria at 10^7^ and 10^6^ cells/mL, but responses to the highest bacterial cell density of 10^8^ cells/mL were not significantly different from responses to MR2A medium alone (*P* > 0.05). Some bacterial isolates significantly attracted gravid females at only a single cell density, namely B4 (10^7^ cells/mL, *P* = 0.0217), B6 (10^6^ cells/mL, *P* = 0.0051), and B7 (10^6^ cells/mL, *P* = 0.0407) (data not shown). *Ae. aegypti* females were repelled by one isolate at a cell density of 10^8^ cells/mL (B11,* P* = 0.0407). Bacterial isolates B8, B9 and B10 did not elicit significant responses to any of the three cell densities tested (Table 3). Among all the isolates tested at three different cell densities, approximately 10 to 21 % of the *Ae. aegypti* females remained free in the test arena.

**Table 3:** Summary of 2-choice sticky screen attraction bioassays in which the attraction of *Ae. aegypti* and *Ae. albopictus* to different bacterial species at different cell densities was tested against MR2A medium, as in Figs. 1 and 2

	*Ae. aegypti*			*Ae. albopictus*		
Isolate	10^6^	10^7^	10^8^	10^6^	10^7^	10^8^
B1	+	++	NS	NS	+	−
B2	+	++	NS	NS	NS	NS
B3	+	+	NS	NS	NS	NS
B4	NS	+	NS	NS	NS	NS
B5	+++	+	NS	NS	++	NS
B6	++	NS	NS	NS	NS	−−
B7	+	NS	NS	NS	++	NS
B8	NS	NS	NS	NS	NS	NS
B9	NS	NS	NS	NS	NS	NS
B10	NS	NS	NS	−	++	NS
B11	NS	NS	−	NS	NS	NS
B12	+	++	NS	NS	NS	NS
B13	+++	++	NS	NS	++	NS
B14	++	+++	NS	+++	NS	−−

NS, no attraction or repellency

Data were analyzed using multinomial regression

Attraction: +, *P* < 0.05; ++, *P* < 0.01; +++, *P* < 0.001

Repellency: −, *P* < 0.05; −− , *P* < 0.01; −−−, *P* < 0.001

### Responses of *Aedes albopictus* to individual bacterial isolates

**Fig. 2:**
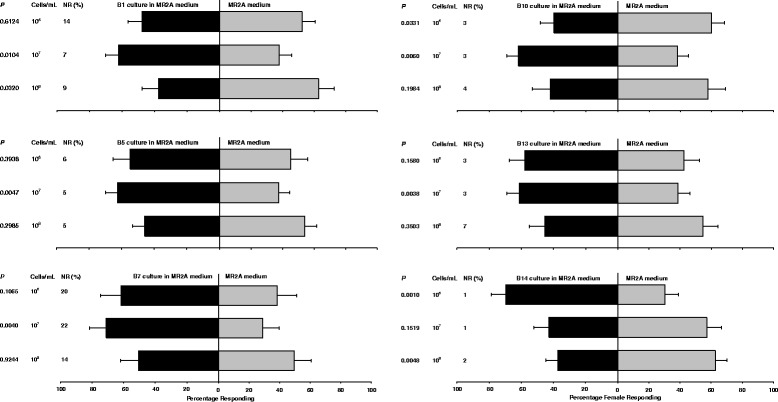
Results of 2-choice sticky screen attraction bioassays in which different bacterial species in liquid medium were tested for their attractiveness to *Ae. albopictus*. Bars show the mean relative attractiveness. Error bars represent half-width of a 95 % confidence intervals of the mean (SEM X 1.96). Each test consisted of 18 assays with 10 gravid females per assay, except isolates B1 and 13 were tested in only 16 assays. Bacterial isolates that did not elicit significant responses to any of the three cell densities are not shown in the figure. NR = not responding

In behavioral assays with single isolates, *Ae. albopictus* females were significantly attracted to 6 of the 14 bacterial isolates, but each isolate was attractive at only a single cell density of either 10^7^ cells/mL (B1, B5, B7, B10 and B13) or 10^6^ cells/mL (B14) (Fig. 2). Gravid females were significantly repelled by isolates B10 (10^6^ cells/mL) and B1 and B14 (10^8^ cells/mL). *Ae. albopictus* females were not attracted or repelled by isolates B2, B3, B4, B8, B9, B11 and B12 at any of the three cell densities tested. Across all these assays, 1 to 22 % of *Ae. albopictus* females did not respond to either treatment and remained free in the test arena.

## Discussion

*Aedes aegypti* and *Ae. albopictus* prefer to lay eggs in water-holding human-made containers. Dark colored containers have been used as ovitraps to mimic oviposition sites. Densities of eggs deposited in ovitraps and numbers of females trapped in sticky ovitraps have been used to predict the size of vector populations [25], to evaluate mosquito control methodologies [26] and to characterize the spatial and temporal activity of both container-inhabiting mosquito species [27–30]. Use of plant-based infusions in ovitraps increases the number of eggs deposited [31–33]. However, the response of gravid mosquitoes to infusions is influenced by the plant species, its biomass in the fermentation process [14, 31, 33, 34], fermentation time [14, 21] and likely other physical and chemical interactions of the microbes with the organic substrate.

Previously, we showed that volatile products of bacterial metabolism in canebrake bamboo leaf infusions were attractive to gravid females of both *Ae. aegypti* and *Ae. albopictus* [21]. Furthermore, changes in the abundance and diversity of bacterial species altered the behavioral response of gravid mosquitoes to these infusions. And finally, a mix of fourteen species of bacteria, isolated and cultured from attractive infusions, elicited significant attraction and oviposition responses from gravid *Ae. aegypti* and *Ae. albopictus* females. Because we modified the composition of the R2A medium, in the present study, we re-evaluated the responses of gravid female mosquitoes of both species to four different cell densities of a mixture of 14 bacterial isolates. We used a sticky-screen bioassay that assessed their attraction to odorants and this assay effectively differentiated the attraction response from subsequent oviposition responses that are significantly affected by contact with water, bacteria and various medium components. Significant attraction responses were exhibited by *Ae. aegypti* to intermediate bacterial densities of 10^7^ and 10^8^ cells/mL, but females were significantly repelled by 10^9^ cells/mL. Similarly, significantly more gravid *Ae. albopictus* were attracted to10^7^ and 10^8^ cells/mL of the mix of 14 bacterial isolates than to the control medium. It is worth highlighting that bacterial isolates produced odorants that attracted gravid mosquitoes when MR2A growth medium was substituted for R2A medium. Thus, results of the present study corroborate our earlier study [21] in which the level of attraction of gravid *Ae. aegypti* and *Ae. albopictus* to canebrake bamboo leaf infusions was correlated with leaf biomass and fermentation time. Similar to our study, gravid females exhibited a dose-dependent reversal of response to bacterial cell density of a mix of 14 bacterial species with the percentage of females trapped on sticky screening increasing from 10^6^ to 10^8^ cells/mL and declining significantly at 10^8^ cells/mL. Similarly, Seenivasagan *et al.* [35] also demonstrated that carboxylic acids can act either as attractants or repellents, depending on their concentration.

Few studies have investigated the relationship between bacteria and oviposition site selection, and often studies did not consider the effects of various bacterial densities, proper untreated controls, and assays that discriminate between behavioral attraction and oviposition. Hasselschwert and Rockett [17] screened different bacterial cultures from the larval habitat of *Culex* mosquitoes and determined that the presence of *Bacillus cereus* and *Pseudomonas aeruginosa* elicited oviposition responses from *Ae. aegypti*. Similarly, Trexler *et al.* [13] showed that individual isolates of *Sphingobacterium multivorum* (from soil-contaminated cotton towels)*, Psychrobacter immobilis* (from larval-rearing water) or an unidentified *Bacillus* species (from oak leaf infusion) inoculated into water elicited higher oviposition responses from gravid *Ae. albopictus* than did water without bacteria. Poonam *et al.* [36] produced cell-free filtrates from pure bacteria cultures and found that gravid *Cx. quinquefasciatus* females oviposited more in certain concentrations of *Bacillus cereus*, *Bacillus thuringiensis* and *Pseudomonas fluorescens* than in tap water. Filtrates of some bacteria (e.g., *Bacillus megaterium*, *Azospirillum brasilense*) failed to stimulate more oviposition than water alone at any concentration, showing some selectivity of the mosquito responses to bacterial culture filtrates [36]. Notably however, different bacteria were grown in different media and the respective medium was not used as control; the medium itself may contain attractants, repellents, as well as oviposition stimulants and deterrents. Finally, Huang *et al.* [37] demonstrated that gravid *An. gambiae* did not exhibit significant oviposition responses to a mixture of bacteria (*Acinetobacter, Aeromonas*, *Bacillus*, *Enterobacter*, *Klebsiella, Pantoea, Pseudomonas,* and *Stenotrophomonas*) originating from a natural larval habitat; bacterial cultures were presented to mosquitoes on agar plates at varying cell densities for the bacterial species. Our study highlights the importance of (a) careful attention to clear behavioral assays that discriminate between behavioral attraction and the outcomes of oviposition behavior, (b) the use of proper untreated controls (in this case, culture medium), (c) testing individual bacterial isolates, and (d) conducting extensive dose–response studies that relate mosquito behavior to various bacterial densities.

When all 14 bacterial isolates were tested together, *Ae. aegypti* females were significantly repelled by 10^9^ cells/mL and approximately 35 % of the females failed to respond and remained free in the test arena. But as the mix of 14 bacterial isolates became significantly attractive to *Ae. aegypti* females at 10^8^ and 10^7^ cells/mL, more females were trapped and fewer females (20-22 %) remained free. Similarly, *Ae. albopictus* females were significantly attracted to 10^8^ and 10^7^ cells/mL of the 14-bacteria mix, and fewer females remained free in the test arenas at these bacteria densities than at either 10^6^ or 10^9^ cells/mL. The present results suggest that when a mixture of isolates containing both attractive and repellent chemicals is tested, more *Ae. aegypti* and *Ae. albopictus* females remain unresponsive. Notably, the numbers of non-responsive mosquitoes were lower when we tested single bacterial isolates.

Interestingly, although both *Ae. aegypti* and *Ae. albopictus* females were attracted to the bamboo leaf infusion and to the mix of 14 bacterial species that we isolated from it [9, 21] (present study), the mosquitoes exhibited vastly different responses to each bacterial species at various cell densities. *Ae. aegypti* females tended to respond to lower bacterial cell densities than *Ae. albopictus* females. Moreover, some bacterial species attracted *Ae. aegypti* but not *Ae. albopictus* females (e.g., B2, B3, B4, B6 and B12), but only B10 attracted *Ae. albopictus* and not *Ae. aegypti* females. Most importantly, some bacterial species, namely B1, B5, B13 and B14, were highly attractive to both *Aedes* species. These bacterial species obviously are important candidates for further investigations and deployment in traps. Our results suggest that not all bacterial species produced the same chemicals cues (attractants or repellents) or amounts of attractants at the same cell density. It is likely that the chemical composition of the oviposition semiochemicals varied across bacterial species, which is the likely cause of differences in the responses of the two mosquito species. Furthermore, it is also possible that variation in bacterial generation time might have resulted in quantitative and/or qualitative differences in chemical cues. Indeed, a recent study [38] described significant differences in the concentration and types of volatile compounds produced by different bacteria grown in the laboratory.

## Conclusions

Our results show that at specific cell densities, some bacteria significantly influence the attraction of gravid *Ae. aegypti* and *Ae. albopictus* females to potential oviposition sites. Whereas some bacteria are highly attractive to females, other bacterial species within the same infusion may be highly repellent, suggesting that the complex leaf infusions or alfalfa infusions that are currently deployed for mosquito control may be inferior to mixes of only selected attractive bacteria. These findings indicate that selected attractive bacteria could be used to exploit the chemotactic orientation behavior of mosquitoes for population control purposes. For example, coupling attractive bacteria with a trap containing a toxicant could be used as the basis for a lure-and-kill management strategy for *Ae. aegypti* and *Ae. albopictus*. Additional research will be needed to find a suitable carrier for sustained release formulations of bacterial species that sustain the desired cell densities.
